# Supplementary material for: A framework for focal and connectomic mapping of transiently disrupted brain function
Source: Commun Biol. 2023 Apr 19;6:430. doi: 10.1038/s42003-023-04787-1 (PMC10115870; doi:10.1038/s42003-023-04787-1)
Supplement: Supplementary file 1 — Supplementary Material [file 42003_2023_4787_MOESM1_ESM.pdf]

Supplementary Table T1. Patient clinical characteristics

Patient	Type	Sex	Handed	Age at onset (years)	Age at ICR (years)	Epilepsy Duration (years)	Side	Language dominance	Imaging abnormality	Ictal onset zone	Pathology (if operated)	ILAE outcome (months)
1	Grid	F	Left	3	27	24	Left	Left	None	Left SFG	FCD iia	1 (39)
2	Grid	F	Right	12	36	24	Left	Left	None	Left mesial SFG	Non-specific	5 (54)
3	Grid	M	Right	6	26	20	Right	Bilateral	None	Right SFG/precentral	FCD iia	4 (33)
4	Grid	F	Right	10	28	18	Right	Left	None	Right SFG	FCD iib	1 (54)
5	Grid	F	Right	8	23	15	Left	Left	Left insula lesion	Left anterior frontal	Gliosis	5 (27)
6	Grid	M	Right	12	20	8	Left	Left	None	Left pre/post central/SMA	Inoperable	N/A
7	Grid+depth	M	Right	16	45	29	Left	Left	None	Not localised	Inoperable	N/A
8	Grid+depth	M	Right	2	21	19	Right	Left	Dysplastic left precentral gyrus	Primary motor area	Inoperable	N/A
9	Grid+depth	M	Right	38	48	10	Left	Left	None	Left SMA	Inoperable	N/A
10	Grid+depth	M	Right	10	23	13	Right	N/a	None	Not localised	Inoperable	N/A
11	Grid+depth	M	Right	6	25	19	Left	Left	Left central gyrus signal change	Right hand sensory motor area	Inoperable	N/A
12	Grid+depth	F	Right	11	21	10	Left	Left	Cerebellar lesion	Left primary motor cortex	Inoperable	N/A
13	Grid+depth	M	Right	8	18	10	Left	Left	Left precentral gyrus tumour	Left paracentral/precentral	Inoperable	N/A
14	Grid+depth	M	Right	6	38	32	Right	Left	Dysplastic right SFG	Right SMA	Gliosis	5 (42)
15	Grid+depth	M	Right	29	41	12	Left	Left	None	Left SMA	Non-specific	5 (12)
16	Grid+depth	F	Right	5	49	44	Left	Left	Dysplastic left IFG	Left IFG	Inoperable	N/A
17	Grid+depth	M	Right	12	32	20	Right	Bilateral	Right parietal dysplasia	Right SPL	FCD iib	1 (54)
18	Grid+depth	M	Right	14	49	35	Left	Left	Resected left MFG	Left SFG and MFG	DNET	3 (19)
19	Depth	M	Right	13	30	17	Left	Left	Non lesional	Left anterior medial frontal	Non-specific	5 (12)
20	Depth	M	Right	15	29	14	Left	Left	Non lesional	Left insula	Inoperable	N/A
21	Depth	M	Right	25	46	21	Right	Bilateral	Non lesional	Right orbito-/inferior frontal	Inoperable	N/A
22	Depth	M	Right	12	30	18	Right	Bilateral	Non lesional	Right mesiofrontal	Awaiting	N/A
23	Depth	M	Right	10	33	23	Right	Left	Non lesional	Right insular	Awaiting	N/A
24	Depth	F	Right	13	46	33	Left	Left	Left frontal dysplasia	Left middle frontal gyrus	Awaiting	N/A
25	Depth	M	Right	7	32	25	Left	Bilateral	Left HS	Left temporal	Awaiting	N/A
26	Depth	M	Equal	1	37	36	Left	Left	Left HS	Left hippocampus	HS	1 (4)
27	Depth	M	Right	23	27	4	Right	Left	None	Right SFG and MFG	Awaiting	N/a
28	Depth	M	Right	5	19	14	Right	Unclear	None	Right SFG	FCD iib	3 (13)
29	Depth	M	Right	1	26	25	Both	Left	None	Right SFG and MFG	Inoperable	N/A
30	Depth	F	Right	6	22	16	Left	N/a	None	Left anterior insula	Inoperable	N/A
31	Depth	M	Right	7	41	34	Left	Left	None	Left insula	Inoperable	N/A
32	Depth	M	Equal	3	44	41	Left	Right	Left frontal dysplasia	Left frontopolar/orbital	Awaiting	N/A
33	Depth	M	Right	11	41	30	Right	Left	Lesion SFG	Right SFG	Inoperable	4 (15)
34	Depth	F	Right	3	34	31	Left	Left	Left frontal dysplasia	Frontal lobe	Inoperable	N/A
35	Depth	M	Right	16	32	16	Right	Left	None	Right orbitofrontal	Gliosis	1 (36)
36	Depth	M	Right	38	68	30	Right	Left	None	Right SMA	Non-specific	1 (60)
37	Depth	M	Left	6	46	40	Right	Left	None	Right orbitofrontal	Normal	1 (6)

Supplementary Table T1. Patient clinical characteristics. Summary of demographics and clinical characteristics of the included patients with stimulations in the medial frontal cortex, based on Trevisi et al. (2018). F = female; M = male; ICR = intracranial recording; FCD = focal cortical dysplasia; SMA = supplementary motor area; HS = hippocampal sclerosis; DNET = dysembryoplastic neuroepithelial tumour; SFG = superior frontal gyrus; SMG =superior medial gyrus; IFG = inferior frontal gyrus; SPL = superior parietal gyrus; ILAE = international league against epilepsy.

Supplementary Table T2: MNI Coordinates

Positive Motor (n = 153)

	x	y	z
1	1	1	42
2	10	−23	51
3	−18	−16	54
4	−39	−3	39
5	1	−30	55
6	2	−10	40
7	3	−1	47
8	−8	14	48
9	−5	−1	60
10	−2	−2	64
11	23	−13	32
12	−2	−12	52
13	−1	−9	57
14	−1	−31	43
15	0	−6	62
16	3	−4	67
17	−1	−21	37
18	−1	−29	49
19	−1	−34	59
20	−1	−40	68
21	0	−22	59
22	0	−21	70
23	0	−17	58
24	0	−7	61
25	1	−45	74
26	5	−39	62
27	19	−20	66
28	0	−8	62
29	0	−13	64
30	1	−3	61
31	2	−4	56
32	2	−8	56
33	2	−14	58
34	3	0	48
35	3	−6	49
36	3	−10	51
37	3	−15	53
38	4	−7	44
39	4	−12	45
40	4	−17	47
41	−7	−12	56
42	−4	11	39
43	−9	9	55
44	−7	17	39
45	−33	−18	51
46	−28	−31	51
47	−14	−18	65
48	−13	−23	66
49	−13	−33	66
50	−13	−36	65
51	−10	−24	68

	x	y	z
52	−9	−19	69
53	−8	−13	57
54	−5	−6	44
55	−4	−3	45
56	−4	2	43
57	−4	−2	41
58	−3	−7	54
59	−3	0	47
60	−3	−5	49
61	0	−30	58
62	0	−6	59
63	0	−1	56
64	2	−1	52
65	4	−9	52
66	−2	−13	69
67	−2	−8	67
68	2	−15	65
69	2	−9	63
70	4	−16	59
71	4	−10	57
72	−32	−24	30
73	−2	9	35
74	−2	12	41
75	−2	−6	50
76	−2	−3	56
77	−2	0	67
78	−2	−10	55
79	−2	−7	66
80	−2	−15	58
81	−2	−13	64
82	−1	−12	69
83	−22	−15	60
84	−5	−22	47
85	0	−8	48
86	0	−22	45
87	−37	−9	55
88	−31	−9	54
89	−25	−9	51
90	−20	−9	48
91	−13	−9	46
92	2	−14	57
93	6	−15	61
94	29	−16	71
95	7	−14	55
96	7	−19	59
97	9	−6	47
98	12	−5	51
99	12	−19	62
100	16	−19	64
101	20	−18	68
102	25	−18	69

	x	y	z
103	25	−18	70
104	29	−18	71
105	6	1	47
106	7	−23	58
107	10	−24	62
108	11	3	52
109	14	−23	66
110	−22	−6	55
111	−21	−23	66
112	−16	−23	64
113	−12	−9	52
114	−7	−11	51
115	−22	−4	65
116	−17	−3	63
117	−12	−2	62
118	−9	−1	61
119	−3	2	57
120	0	4	55
121	−33	2	50
122	−22	1	49
123	−17	1	49
124	−11	1	50
125	−6	1	50
126	−1	1	50
127	5	1	49
128	10	1	48
129	0	−21	64
130	1	−32	63
131	2	−27	61
132	4	−18	56
133	4	−29	56
134	4	−36	53
135	5	13	27
136	5	16	31
137	5	5	34
138	5	9	39
139	5	−19	51
140	5	−23	54
141	5	−31	51
142	6	9	31
143	6	1	38
144	6	−21	47
145	6	−26	50
146	−1	−28	62
147	3	−37	67
148	0	−12	45
149	0	−12	57
150	0	−12	62
151	4	−11	68
152	9	−11	69
153	14	−11	70

Negative Motor (n = 41)

	x	y	z
1	1	1	42
2	2	8	55
3	−8	14	48
4	5	2	66
5	−3	−14	47
6	1	2	59
7	2	1	54
8	3	0	48
9	3	−6	49
10	4	−7	44
11	−7	−12	56
12	8	42	44
13	−8	−13	57
14	−5	−6	44
15	−4	−2	41
16	−3	0	47
17	−3	−5	49
18	−2	−8	58
19	−1	−3	55
20	−6	18	46
21	2	−1	52
22	4	−9	52
23	−32	−24	30
24	−19	−2	53
25	−4	−7	48
26	0	−8	48
27	1	17	48
28	5	19	52
29	8	12	37
30	−8	13	54
31	−3	12	53
32	21	5	64
33	−27	−5	55
34	−22	−6	55
35	−17	9	63
36	−17	−8	53
37	−12	8	59
38	−9	7	56
39	−6	5	52
40	−2	4	48
41	0	3	42

Sensory (n = 46)

	x	y	z
1	3	6	46
2	10	−23	51
3	−39	−3	39
4	2	8	55
5	−8	14	48
6	−2	−2	64
7	−4	−16	41
8	−2	14	53
9	1	18	57
10	−7	−12	56
11	−13	−40	65
12	−3	−9	52
13	−5	−9	41
14	−5	0	37
15	2	−3	61
16	4	−5	55
17	4	1	53
18	−2	5	40
19	−2	0	44
20	−2	2	50
21	4	−9	38
22	−2	−9	41
23	−8	−9	44
24	5	13	49
25	7	14	52
26	11	14	56
27	4	−9	38
28	7	−7	43
29	14	−3	55
30	16	−2	58
31	29	−18	71
32	−7	−11	51
33	−28	2	49
34	5	13	27
35	−2	−32	69
36	−1	−26	66
37	3	−22	59
38	5	−31	51
39	4	−29	56
40	2	−27	61
41	4	−36	53
42	3	−34	58
42	−1	1	40
44	−1	−6	45
45	−1	−20	58
46	0	−12	51

Speech (n = 46)

	x	y	z
1	−1	23	26
2	3	20	43
3	2	8	55
4	−8	14	48
5	5	2	66
6	7	2	65
7	13	−9	68
8	8	42	44
9	0	6	51
10	−1	−3	55
11	−2	−8	58
12	−3	−9	52
13	−5	−5	40
14	−5	5	35
15	−4	−3	45
16	−4	2	43
17	−4	−2	41
18	−3	0	47
19	−3	−5	49
20	2	−1	52
21	−1	5	64
22	−2	−8	60
23	−2	−7	66
24	−2	−13	64
25	−1	−12	69
26	−1	−10	73
27	0	−18	68
28	1	−17	73
29	2	−16	76
30	−19	−2	53
31	−23	−1	54
32	−27	0	56
33	−32	1.3	59
34	1	6	60
35	−4	6	64
36	−8	6	67
37	−13	5	70
38	−18	5	70
39	1	17	48
40	5	19	52
41	8	12	37
42	−3	12	53
42	−8	13	54
44	0	3	42
45	−12	8	59
46	6	9	31

No Response (n = 243)

	x	y	z
1	0	57	22
2	0	52	31
3	0	46	39
4	0	41	48
5	0	34	57
6	−2	29	48
7	−2	20	55
8	−2	10	62
9	−3	9	53
10	0	11	58
11	3	12	62
12	7	13	65
13	−3	14	49
14	0	15	55
15	4	17	59
16	9	18	62
17	−2	19	46
18	1	20	52
19	5	22	56
20	9	22	58
21	−1	23	43
22	1	26	48
23	5	27	52
24	9	28	55
25	−1	28	40
26	3	31	45
27	6	32	48
28	10	33	51
29	0	34	37
30	3	35	42
31	7	37	45
32	11	39	47
33	0	39	35
34	4	40	38
35	8	42	41
36	12	43	43
37	1	45	32
38	5	46	36
39	9	48	39
40	14	−49	40
41	7	5	56
42	−11	24	56
43	1	20	43
44	0	24	48
45	−2	27	53
46	1	24	39
47	0	27	44
48	−2	31	49
49	1	26	36
50	0	30	41
51	−2	34	46

	x	y	z
52	1	29	31
53	0	33	36
54	−2	37	41
55	1	34	28
56	0	38	33
57	−1	41	38
58	2	37	24
59	1	41	30
60	0	45	34
61	2	40	21
62	1	45	26
63	0	48	30
64	2	44	17
65	2	49	22
66	1	52	27
67	−5	30	48
68	−1	−15	69
69	2	−13	69
70	6	−8	68
71	7	−2	70
72	−5	−19	35
73	−2	16	44
74	−2	19	49
75	2	22	53
76	4	25	57
77	−3	11	48
78	4	20	60
79	0	−16	69
80	0	−13	67
81	0	−13	56
82	0	−6	66
83	4	−2	42
84	−60	−62	29
85	−55	−59	37
86	−50	−55	44
87	−46	−53	52
88	−40	−50	60
89	−33	−46	67
90	−24	−43	72
91	−14	−39	72
92	−63	−52	27
93	−60	−47	35
94	−55	−44	45
95	−51	−42	54
96	−44	−38	61
97	−36	−35	67
98	−28	−32	72
99	−18	−28	73
100	−65	−40	23
101	−64	−37	34
102	−59	−33	45

	x	y	z
103	−54	−30	53
104	−47	−27	61
105	−38	−23	66
106	−29	−20	70
107	−21	−16	71
108	−67	−32	19
109	−63	−28	30
110	−60	−23	41
111	−55	−19	50
112	−49	−16	59
113	−41	−13	63
114	−31	−10	66
115	−22	−7	68
116	−2	39	42
117	−9	40	22
118	−11	46	39
119	8	44	28
120	−15	−14	64
121	−1	52	25
122	−5	47	15
123	−1	47	28
124	−5	43	18
125	0	41	34
126	−2	39	29
127	−5	37	25
128	−5	34	19
129	−1	27	44
130	−3	25	38
131	0	22	46
132	−5	19	34
133	0	17	47
134	−5	14	36
135	−3	9	37
136	−4	3	40
137	−1	1	53
138	−5	−11	46
139	−5	−8	47
140	−4	6	41
141	−2	3	49
142	1	4	54
143	−3	4	45
144	−2	−1	65
145	4	11	48
146	5	11	41
147	5	13	35
148	3	17	50
149	3	18	43
150	4	19	36
151	2	23	51
152	2	23	44
153	3	24	37

	x	y	z
154	0	28	51
155	0	28	44
156	1	29	38
157	−6	26	50
158	−4	39	28
159	0	46	38
160	−4	43	25
161	0	50	34
162	−4	46	21
163	0	52	31
164	−4	50	18
165	0	57	27
166	2	67	5
167	3	59	26
168	1	63	3
169	1	61	15
170	1	56	26
171	1	52	39
172	1	46	53
173	1	39	62
174	2	13	32
175	−2	17	36
176	−2	19	43
177	−2	21	50
178	−2	14	47
179	−2	17	54
180	−2	7	46
181	−2	8	52
182	−2	12	58
183	−2	5	57
184	−2	6	62
185	−2	−1	61
186	−1	−5	70
187	−1	−20	62
188	1	20	33
189	−1	19	39
190	−14	19	67
191	17	22	64
192	2	12	36
193	33	15	45
194	13	16	60
195	16	17	64
196	−12	13	55
197	14	4	56
198	18	5	60
199	−21	10	65
200	−24	−22	69
201	0	32	43
202	−4	35	46
203	−7	39	47

	x	y	z
204	−2	51	16
205	−2	48	27
206	0	42	37
207	3	38	46
208	5	33	54
209	9	28	61
210	4	20	35
211	5	12	36
212	4	17	40
213	4	13	43
214	6	5	42
215	5	9	46
216	4	40	6
217	4	40	19
218	4	40	31
219	4	35	42
220	0	31	50
221	−1	−14	52
222	0	−12	39
223	−2	−37	36
224	−1	−47	31
225	−2	−40	41
226	−2	−49	36
227	−2	−41	46
228	−2	−51	40
229	−2	−43	52
230	−2	−53	45
231	−1	−45	55
232	−1	−54	50
233	2	−47	60
234	1	−56	54
235	5	−48	64
236	5	−56	58
237	10	−48	65
238	9	−56	63
239	3	51	7
240	3	47	18
241	3	43	30
242	3	39	41
243	−1	35	54

Supplementary Table T3. Statistical results of local disruptive mapping

Behaviour	t-statistic	*p* value (FWE corrected)	MNI Coordinates x	y	z	Anatomical Location
Positive Motor	7.94	<0.000	4	−10	60	Supplementary motor area
	3.92	0.049	12	−15	66	Precentral gyrus (medial segment)
Negative Motor	7.15	<0.000	0	−4	48	Supplementary motor area
Sensory	5.91	<0.000	−2	−6	34	Middle cingulate gyrus
	5.21	<0.000	9	−33	56	Precentral gyrus (medial segment)
Speech	5.36	<0.000	−10	6	66	Superior frontal gyrus
	5.25	<0.000	−3	−14	76	Superior frontal gyrus
	3.96	0.042	−22	3	57	Pre-supplementary motor area
No Response	5.21	<0.000	2	18	38	Middle cingulate gyrus
	4.36	0.010	−8	32	44	Superior frontal gyrus (medial segment)

Supplementary Table T3. Statistical results of local disruptive mapping. For each of the behavioural effects, the t-statistics, *p* values (FWE corrected) and MNI coordinates of the peak-level voxels are provided, as well as the nearest grey-matter anatomical location.

Supplementary Table T4. Statistical results of connective disruptive mapping

Behaviour	t-statistic	*p* value (FWE corrected)	MNI Coordinates x	y	z	Anatomical Location
Positive Motor	10.17	<0.000	0	−74	−42	Fronto-parietal cerebellar network
	8.74	<0.000	4	−32	66	Precentral gyrus (medial segment)
	7.74	<0.000	14	−88	30	Superior occipital gyrus
	7.62	<0.000	0	0	2	Thalamus
	7.43	<0.000	−16	−50	60	Superior parietal lobule
	6.13	<0.000	−18	−86	28	Superior occipital gyrus
	5.77	<0.000	32	−22	16	Posterior insula
	5.64	0.001	−18	−26	−4	Thalamus
	5.44	0.001	20	−16	20	Caudate
	5.33	0.002	−16	−2	60	Superior frontal gyrus
	5.00	0.011	26	−4	0	Pallidum
	4.90	0.016	42	−70	−36	Sensory-motor foot cerebellar network
	4.88	0.017	−30	−64	−60	Sensory-motor hand cerebellar network
	4.63	0.048	48	−8	14	Central operculum
Negative Motor	6.15	<0.000	−8	−60	64	Superior parietal lobule
	6.00	<0.000	0	−10	52	Supplementary motor area
	5.92	<0.000	0	18	50	Supplementary motor area
	5.71	<0.000	−10	−76	−36	Dorsal attention cerebellar network
	5.57	0.001	−14	8	50	Middle frontal gyrus
	5.45	0.001	−16	0	72	Superior frontal gyrus
	5.21	0.004	−20	−64	−46	Fronto-parietal cerebellar network
	5.16	0.005	20	−12	66	Precentral gyrus
	5.12	0.006	2	−72	−18	Fronto-parietal cerebellar network
	4.94	0.013	0	−34	68	Precentral gyrus (medial segment)
	4.90	0.016	16	−2	52	Superior frontal gyrus
	4.88	0.017	4	−74	46	Precuneus
Sensory	5.48	0.001	−4	−18	68	Precentral gyrus (medial segment)
	5.40	0.002	−2	−40	−70	Medulla
	4.96	0.012	0	−70	−22	Fronto-parietal cerebellar network
	4.87	0.017	−6	−2	0	Thalamus
	4.80	0.023	0	−6	46	Middle cingulate gyrus
No Response	9.05	<0.000	0	36	42	Superior frontal gyrus (medial segment)
	8.26	<0.000	22	−84	−40	Dorsal attention cerebellar network
	7.60	<0.000	0	−54	14	Precuneus
	6.49	<0.000	20	−60	30	Precuneus
	5.60	0.001	−36	−76	38	Angular gyrus
	5.54	0.001	24	12	44	Middle frontal gyrus
	4.89	0.017	−14	22	-24	Medial orbital gyrus

Supplementary Table T4. Statistical results of connective disruptive mapping. For each of the behavioural effects, the t-statistics, *p* values (FWE-corrected) and MNI coordinates of the peak-level voxels are provided, as well as the nearest grey-matter anatomical location.

Supplementary Table T5. Mean and 95% confidence intervals of classification parameters of 100 bootstraps of the test set

Behaviour	Approach	True Positives	False Negatives	False positives	True Negatives	Accuracy	Balanced Accuracy	Precision	True Positive Rate (TPR)	False Positive Rate (FPR)	Area Under Curve (AUC)
Positive Motor	SPM	14.71<br>[11.71, 17.71]	16.17<br>[12.83, 19.51]	5.63<br>[3.37, 7.89]	59.09<br>[54.86, 63.32]	0.77<br>[0.73, 0.81]	0.69<br>[0.65, 0.74]	0.73<br>[0.63, 0.82]	0.48<br>[0.39, 0.57]	0.09<br>[0.05, 0.12]	0.87<br>[0.86, 0.8]
	ROI	10.88<br>[8.25, 13.51]	19.94<br>[16.61, 23.27]	3.7<br>[2.19, 5.21]	60.43<br>[56.61, 64.25]	0.75<br>[0.72, 0.79]	0.65<br>[0.61, 0.69]	0.75<br>[0.65, 0.84]	0.35<br>[0.28, 0.43]	0.06<br>[0.03, 0.08]	0.81<br>[0.80, 0.2]
Negative Motor	SPM	2.45<br>[1.26, 3.64]	5.85<br>[3.9, 7.8]	7.42<br>[4.58, 10.26]	79.9<br>[76.97, 82.83]	0.86<br>[0.83, 0.89]	0.61<br>[0.54, 0.67]	0.26<br>[0.13, 0.39]	0.3<br>[0.17, 0.43]	0.08<br>[0.05, 0.12]	0.82<br>[0.79, 0.83]
	ROI	1.13<br>[0, 2.36]	7.17<br>[4.73, 9.61]	6.7<br>[0.59, 12.81]	79.93<br>[73.72, 86.14]	0.85<br>[0.8, 0.91]	0.53<br>[0.48, 0.58]	N/A	0.14<br>[0, 0.29]	0.08<br>[0.01, 0.15]	0.72<br>[0.71, 0.74]
Sensory	SPM	0.98<br>[0.14, 1.82]	8.49<br>[6.25, 10.73]	3.26<br>[1.19, 5.33]	83.03<br>[80.03, 86.03]	0.88<br>[0.85, 0.91]	0.53<br>[0.49, 0.58]	N/A	0.1<br>[0.02, 0.19]	0.04<br>[0.01, 0.06]	0.76<br>[0.75, 0.77]
	ROI	0<br>[0, 0]	9.47<br>[7.07, 11.87]	0<br>[0, 0]	85<br>[83.07, 88.21]	0.9<br>[0.88, 0.93]	0.5<br>[0.5, 0.5]	N/A	0<br>[0, 0]	0<br>[0, 0]	0.65<br>[0.63, 0.67]
Speech	SPM	1.41<br>[0.47, 2.35]	7.48<br>[4.97, 9.99]	2.47<br>[0.95, 4.53]	83.99<br>[81.17, 86.81]	0.89<br>[0.86, 0.92]	0.57<br>[0.51, 0.62]	N/A	0.16<br>[0.05, 0.28]	0.03<br>[0.05, 0.28]	0.8<br>[0.79, 0.82]
	ROI	0<br>[0, 0]	8.89<br>[6.32, 11.46]	0;14<br>[0, 1.53]	85.95<br>[83.07, 88.83]	0.9<br>[0.87, 0.94]	0.5<br>[0.5, 0.5]	N/A	0<br>[0, 0]	0<br>[0, 0]	0.65<br>[0.63, 0.66]

Supplementary Table T5. Mean and Standard Deviation of Classification Parameters of 100 bootstraps of the test set. The mean the standard deviation (± 1 SD in squared brackets) of the performance metrics are given for each behaviour, for both the SPM and ROI classifiers. As can be appreciated, the SPM classifier outperforms the ROI classifier for all the standard classification metrics (i.e., accuracy, balanced accuracy, precision, true positive rate, false positive rate, and AUC).

Supplementary Figure F1: Local disruptive mapping of behaviour

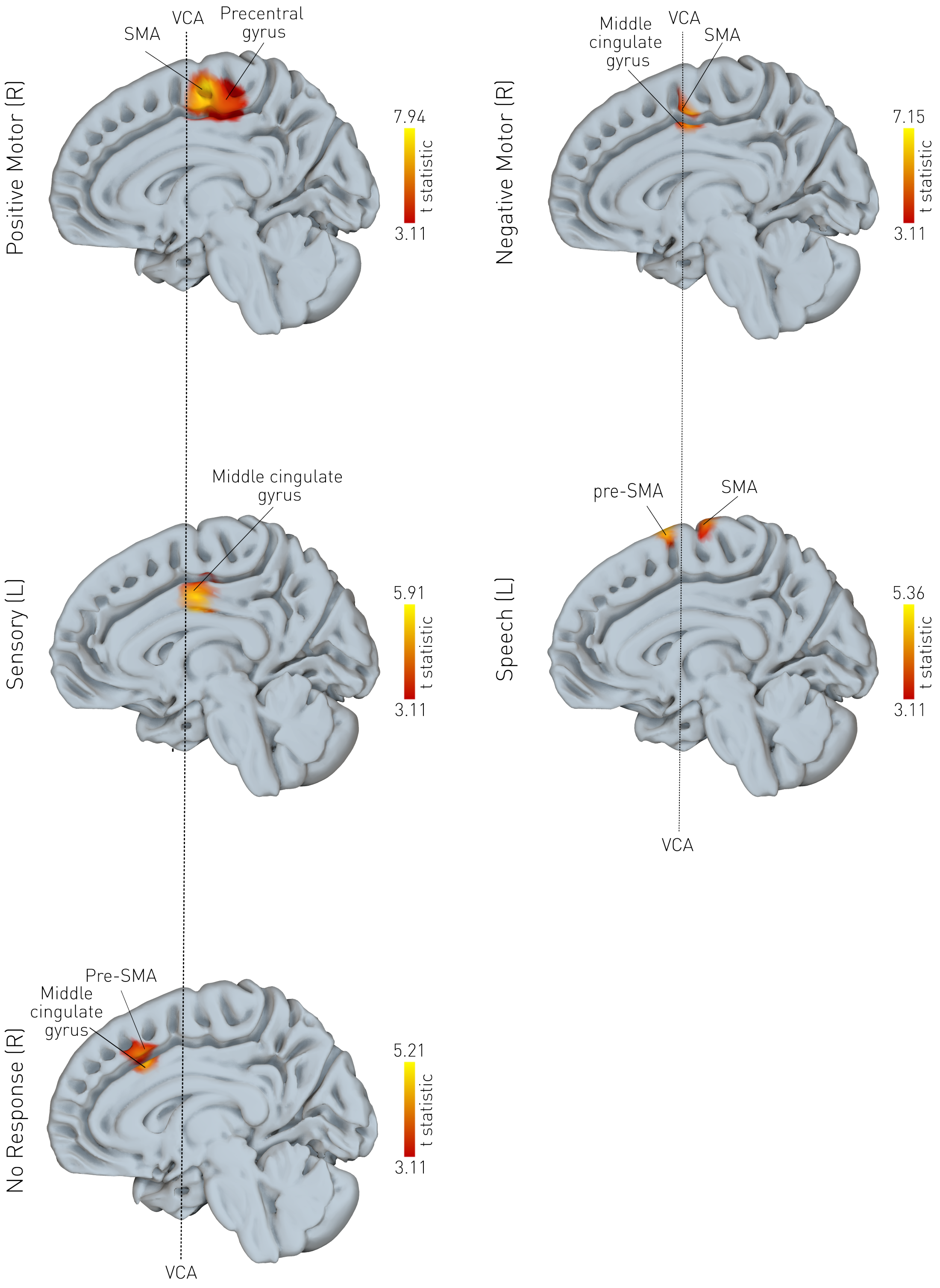

Supplementary Figure F1. Local disruptive mapping of behaviour. For each MNI voxel, a planned t-contrast was performed. Only voxels surviving the $p < 0.001$ uncorrected threshold are shown, overlaid on the mid-sagittal plane, where higher t-statistics (brighter colour) represent a stronger association between the electrode density value and the observed behaviour. R = right; L = left; (pre-)SMA = supplementary motor area.

Supplementary Figure F2: Connective disruptive mapping of behaviour

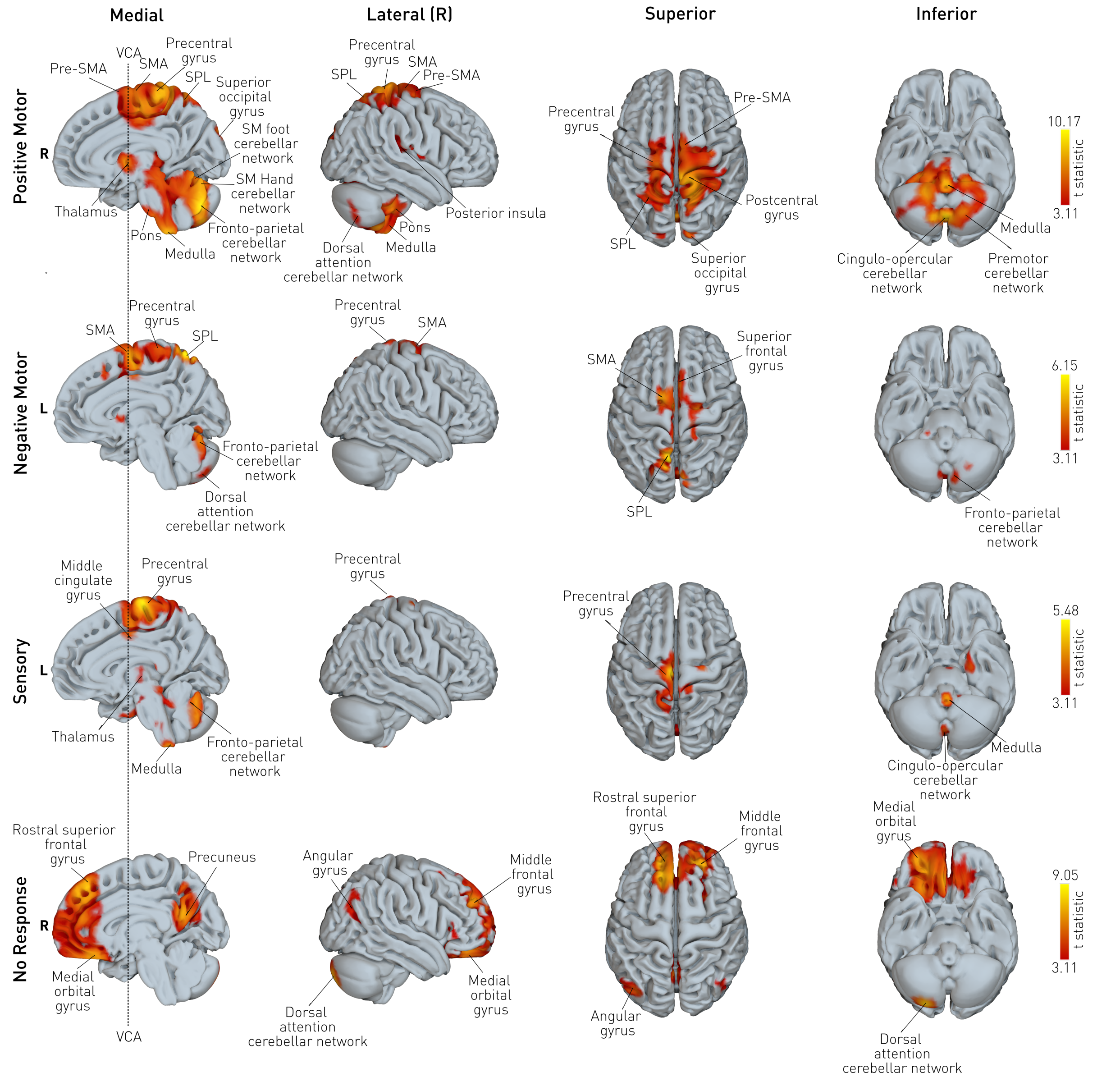

Supplementary Figure F2. Connective disruptive maps of behaviour. For each MNI voxel, a planned t-contrast was performed. Only voxels surviving the $p < 0.001$ uncorrected threshold are shown, overlaid on the mid-sagittal, lateral, superior, and inferior planes, where higher t-statistics (brighter colour) represent a stronger association between the connectivity value and the observed behaviour. Cerebellar subregions are labelled with reference to a priori known cortical network associations (Marek et al., 2018). R = right; L = left; SMA = supplementary motor area; SPL = superior parietal lobule, SM = sensorimotor).

Supplementary Figure F3: Sensitivity Analysis of Local disruptive mapping of behaviour

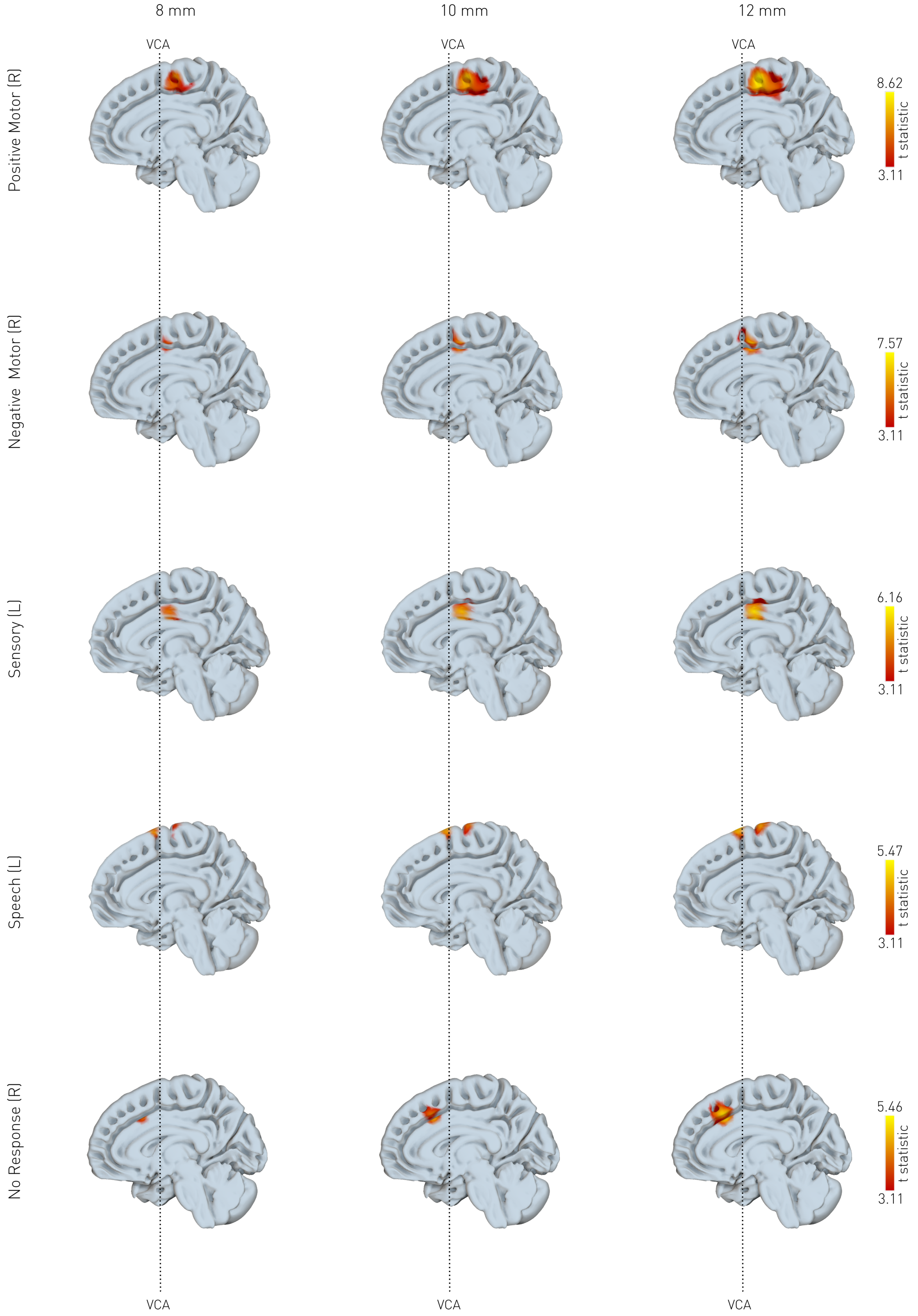

Supplementary Figure F3. Sensitivity Analysis of Local disruptive mapping of behaviour. For each MNI voxel, a planned t-contrast was performed. Only voxels surviving the $p < 0.001$ uncorrected threshold are shown, overlaid on the mid-sagittal plane, where higher t-statistics (brighter colour) represent a stronger association between the electrode density value and the observed behaviour. R = right; L = left; Analysis was replicated using different kernel sizes (8, 10, 12 mm isotropic) showing the results are not dependent on the chosen kernel size of 10 mm.
