# Supplementary material for: A framework for focal and connectomic mapping of transiently disrupted brain function
Source: Commun Biol. 2023 Apr 19;6:430. doi: 10.1038/s42003-023-04787-1 (PMC10115870; doi:10.1038/s42003-023-04787-1)
Supplement: Supplementary file 2 — Description of Additional Supplementary Files [file 42003_2023_4787_MOESM2_ESM.docx]

**Description of Additional Supplementary Files**

**File name:** Supplementary Data

**Description:** The raw data behind the predictive analysis presented in the paper (fig 5) and supplementary table T5.
