# Supplementary material for: A framework for focal and connectomic mapping of transiently disrupted brain function
Source: Commun Biol. 2023 Apr 19;6:430. doi: 10.1038/s42003-023-04787-1 (PMC10115870; doi:10.1038/s42003-023-04787-1)
Supplement: Supplementary file 4 — Reporting Summary [file 42003_2023_4787_MOESM4_ESM.pdf]

nature portfolio

Corresponding author(s): Michael Elmalem

Last updated by author(s): Mar 23, 2023

Reporting Summary

Nature Portfolio wishes to improve the reproducibility of the work that we publish. This form provides structure for consistency and transparency in reporting. For further information on Nature Portfolio policies, see our Editorial Policies and the Editorial Policy Checklist.

Statistics

For all statistical analyses, confirm that the following items are present in the figure legend, table legend, main text, or Methods section.

n/a	Confirmed	
☐	☒	The exact sample size (*n*) for each experimental group/condition, given as a discrete number and unit of measurement
☐	☒	A statement on whether measurements were taken from distinct samples or whether the same sample was measured repeatedly
☐	☒	The statistical test(s) used AND whether they are one- or two-sided<br>*Only common tests should be described solely by name; describe more complex techniques in the Methods section.*
☐	☒	A description of all covariates tested
☐	☒	A description of any assumptions or corrections, such as tests of normality and adjustment for multiple comparisons
☐	☒	A full description of the statistical parameters including central tendency (e.g. means) or other basic estimates (e.g. regression coefficient) AND variation (e.g. standard deviation) or associated estimates of uncertainty (e.g. confidence intervals)
☐	☒	For null hypothesis testing, the test statistic (e.g. *F*, *t*, *r*) with confidence intervals, effect sizes, degrees of freedom and *P* value noted<br>*Give P values as exact values whenever suitable.*
☒	☐	For Bayesian analysis, information on the choice of priors and Markov chain Monte Carlo settings
☒	☐	For hierarchical and complex designs, identification of the appropriate level for tests and full reporting of outcomes
☒	☐	Estimates of effect sizes (e.g. Cohen's *d*, Pearson's *r*), indicating how they were calculated

Our web collection on statistics for biologists contains articles on many of the points above.

Software and code

Policy information about availability of computer code

Data collection	N/A
Data analysis	SPM12 (https://www.fil.ion.ucl.ac.uk/spm/software/spm12/) running on Matlab 2022b.

For manuscripts utilizing custom algorithms or software that are central to the research but not yet described in published literature, software must be made available to editors and reviewers. We strongly encourage code deposition in a community repository (e.g. GitHub). See the Nature Portfolio guidelines for submitting code & software for further information.

Data

Policy information about availability of data

All manuscripts must include a data availability statement. This statement should provide the following information, where applicable:

- Accession codes, unique identifiers, or web links for publicly available datasets
- A description of any restrictions on data availability
- For clinical datasets or third party data, please ensure that the statement adheres to our policy

Coordinate and behavioural data are available from the corresponding authors on request by email. The raw imaging and video data is derived from clinical studies not licensed for public dissemination by the host institution.

Human research participants

Policy information about studies involving human research participants and Sex and Gender in Research.

Reporting on sex and gender	We use the terms female and male. We describe the analysis of irrevocably anonymized unselected clinical data collected in the course of routine clinical care and have no control over the demographic composition of the cohort.
Population characteristics	Full demographic and diagnostic details are reported in Supplementary Table 1.
Recruitment	This is an analysis of irrevocably anonymized unselected clinical data collected in the course of routine clinical care and reflects the composition of the clinical service.
Ethics oversight	This is a reanalysis of previously published data, with ethical details devolved from the source study.

Note that full information on the approval of the study protocol must also be provided in the manuscript.

Field-specific reporting

Please select the one below that is the best fit for your research. If you are not sure, read the appropriate sections before making your selection.

☒ Life sciences ☐ Behavioural & social sciences ☐ Ecological, evolutionary & environmental sciences

For a reference copy of the document with all sections, see nature.com/documents/nr-reporting-summary-flat.pdf

Life sciences study design

All studies must disclose on these points even when the disclosure is negative.

Sample size	This is a methodological study introducing a novel method of focal and connective brain mapping with disruptive point stimulation data. No sample size calculation is possible in this context.
Data exclusions	None.
Replication	This is a methodological study introducing a novel method of focal and connective brain mapping with disruptive point stimulation data. The data used to demonstrate its application has been previously analysed with a different method. To the extent to which empirical conclusions are drawn they represent a replication.
Randomization	We use unselected data gathered in the course of routine clinical care. Neither randomisation nor control groups are possible.
Blinding	This is methodological study with no allocation to experimental conditions. The empirical validation involves no priors on the spatial results that could conceivably motivate researcher bias.

Reporting for specific materials, systems and methods

We require information from authors about some types of materials, experimental systems and methods used in many studies. Here, indicate whether each material, system or method listed is relevant to your study. If you are not sure if a list item applies to your research, read the appropriate section before selecting a response.

Materials & experimental systems

n/a	Involved in the study	
☒	☐	Antibodies
☒	☐	Eukaryotic cell lines
☒	☐	Palaeontology and archaeology
☒	☐	Animals and other organisms
☒	☐	Clinical data
☒	☐	Dual use research of concern

Methods

n/a	Involved in the study	
☒	☐	ChIP-seq
☒	☐	Flow cytometry
☒	☐	MRI-based neuroimaging
